# Pegylated Liposomal Doxorubicin, Docetaxel, and Trastuzumab as Neoadjuvant Treatment for HER2-Positive Breast Cancer Patients: A Phase II and Biomarker Study

**DOI:** 10.3389/fonc.2022.909426

**Published:** 2022-07-08

**Authors:** Haoqi Wang, Yuntao Li, Yixin Qi, Erbao Zhao, Xiangshun Kong, Chao Yang, Qiqi Yang, Chengyuan Zhang, Yueping Liu, Zhenchuan Song

**Affiliations:** ^1^Breast Center, Fourth Hospital of Hebei Medical University, Key Laboratory for Breast Cancer Molecular Medicine of Hebei Province, Shijiazhuang, China; ^2^Department of Breast Center, Shanxi Cancer Hospital, Taiyuan, China; ^3^Department of Breast Surgery, Xingtai People’s Hospital, Xingtai, China; ^4^Pathology Department, Fourth Hospital of Hebei Medical University, Hebei Province Key Laboratory of Breast Cancer Molecular Medicine, Shijiazhuang, China

**Keywords:** HER2-positive breast cancer, neoadjuvant treatment, pegylated liposomal doxorubicin, trastuzumab, efficacy, safety, biomarker

## Abstract

**Background:**

Combined neoadjuvant chemotherapy with trastuzumab and pertuzumab is the standard regimen for human epidermal growth receptor 2 (HER2)-positive breast cancer (BC). However, pertuzumab is not available because it is not on the market or covered by medicare in some regions or poor economy. Anthracyclines and taxanes are cornerstones in BC chemotherapy, and their combination contributes to satisfactory efficiency in neoadjuvant settings. Nonetheless, concomitant administration of trastuzumab and an anthracycline is generally avoided clinically due to cardiotoxicity. Pegylated liposomal doxorubicin (PLD) is less cardiotoxic compared with traditional anthracyclines. Here, we conducted this prospective study to evaluate the efficacy, safety, and potential biomarkers for PLD plus trastuzumab and docetaxel as neoadjuvant treatment in HER2-positive BC.

**Patients and Methods:**

Patients with stage II or III HER2-positive BC were recruited in this multicenter, open-label, single-arm, phase II study. Eligible patients were given 6 cycles of PLD plus docetaxel and trastuzumab. Primary endpoint was total pathological complete response (tpCR, ypT0/is ypN0). Secondary endpoints were breast pathological complete response (bpCR, ypT0/is), objective response rate (ORR), operation rate, breast-conserving surgery rate, and safety. Metadherin (MTDH), glutaminyl-peptide cyclotransferase (QPCT), topoisomerase II alpha (TOP2A), programmed death ligand 1 (PD-L1), and tumor-infiltrating lymphocytes (TILs) were evaluated in BC tissues pre-neoadjuvant for potential biomarkers.

**Results:**

Between March 2019 and February 2021, 54 patients were enrolled, 50 were included in the analysis, and 35 (70.0%) completed 6 cycles of neoadjuvant treatment. Forty-nine (98.0%) patients underwent surgery with a breast-conserving rate of 44.0%. The tpCR rate, bpCR rate, and ORR were 48.0% (95% CI, 33.7%–62.6%), 60.0% (95% CI, 45.2%–73.6%), and 84.0% (95% CI, 70.9%–92.8%), respectively. tpCR was associated with MTDH (*p* = 0.002) and QPCT (*p* = 0.036) expression but not with TOP2A (*p* = 0.75), PD-L1 (*p* = 0.155), or TILs (*p* = 0.76). Patients with HR-negative status were more likely to achieve bpCR compared with those with HR-positive status (76.2% vs. 48.3%, *p* = 0.047). Grade ≥3 adverse events occurred in 38.0% of patients. Left ventricular ejection fraction decline by ≥10% was reported in 18.0% of patients, and no patient experienced congestive heart failure.

**Conclusions:**

PLD plus docetaxel and trastuzumab might be a potential neoadjuvant regimen for HER2-positive BC with a high tpCR rate and manageable tolerability. MTDH and QPCT are potential predictive markers for tpCR.

## Introduction

Breast cancer (BC) has been the most common malignancy for women worldwide in terms of both morbidity and mortality (1). As a kind of systemic treatment before surgery, neoadjuvant chemotherapy (NAC) has become the preferred treatment for patients with locally advanced BC (2). Human epidermal growth receptor 2 (HER2) is positive in about 20%–25% of breast tumors (3). Although HER2-positive breast tumors are associated with aggressive phenotypes and poor prognosis, they are highly sensitive to some chemotherapeutic agents such as anthracyclines or taxanes (4–6). Previous studies have displayed a high pathological complete response (pCR) rate in neoadjuvant treatment with combined application of anthracyclines and taxane (7). Therefore, anthracycline and taxane-based combination chemotherapy is currently considered to be the standard neoadjuvant regimen for HER2-positive BC (8). However, long-term follow-up data showed that around 15%–24% of patients with BC will still experience disease recurrence—even death (9). In this regard, exploring optimal regimens is crucial for patients with BC to obtain longer survival.

Trastuzumab, the first anti-HER2 monoclonal antibody, was approved by the Food and Drug Administration for HER2-positive BC in 1998 (10). Since the advent of trastuzumab, the outcomes of patients with HER2-positive BC have been greatly improved (11–13). Trastuzumab combined with pertuzumab is the preferred treatment in the HER2-positive neoadjuvant setting. However, pertuzumab is not available because it is not on the market or covered by Medicare in some regions or poor economy. A preclinical study has shown that there are additive interactions between trastuzumab and doxorubicin and synergistic interaction between trastuzumab and docetaxel (14). However, the concurrent use of trastuzumab and an anthracycline is clinically avoided on account of synergetic cardiotoxicity (15).

To overcome the cardiotoxicity and improve the penetration of doxorubicin, liposomal doxorubicin (LD) has been developed (16). Compared with conventional doxorubicin, LD offers a significant reduction in cardiotoxicity while preserving its antitumor efficacy for metastatic BC (17, 18). Therefore, LD can serve as an alternative for traditional anthracyclines in the neoadjuvant setting for HER2-positive BC. Several phase II clinical trials have confirmed that LD plus docetaxel and trastuzumab as neoadjuvant treatment is active in HER2-positive BC and entails a favorable cardiotoxicity profile (19, 20). Pegylated liposomal doxorubicin (PLD) is a formulation of doxorubicin encapsulated in about 100-nm vesicles with a lipophilic surface that is coated with hydrophilic polyethylene glycol (21). Although both LD and PLD show a preferential uptake in tumor tissue, mononuclear phagocytic system uptake is avoided by PLD, resulting in increased circulation time (22, 23). Thus far, no studies have assessed the combination of PLD plus trastuzumab and docetaxel as neoadjuvant treatment for patients with HER2-positive BC. For this purpose, a multicenter, open-label, single-arm, phase II study was designed to assess the efficacy and safety of PLD plus trastuzumab and docetaxel for the neoadjuvant treatment of patients with stage II or III HER2-positive BC for the first time.

## Methods

### Study Design

This study was a multicenter, open-label, single-arm, phase II study conducted in 3 hospitals in China. Patients with stage II or III HER2-positive BC were recruited between March 2019 and February 2021. This trial was registered in the Chinese Clinical Trial Registry (number ChiCTR1900021473) and was done in conformance with Good Clinical Practice guidelines and the Declaration of Helsinki. The implementation and modification of the protocol were approved by the ethics committee of the Fourth Hospital of Hebei Medical University. All patients provided written informed consents.

### Patient Eligibility

Treatment-naive cases aged 18–70 years with histologically verified invasive HER2-positive BC staging II–III were eligible. Patients were required to have a Karnofsky performance status (KPS) score of 80–100, at least one assessable target lesion based on Response Evaluation Criteria in Solid Tumours (RECIST) 1.1, and left ventricular ejection fraction (LVEF) ≥55%. Patients with adequate organ function according to local laboratory examination were also included. Patients who had a history of other malignancies within 5 years (other than cured cervix carcinoma *in situ* or basal cell carcinoma of the skin), with involved supraclavicular or internal mammary lymph nodes, or other conditions that researchers considered inappropriate for participation were ineligible for this study. Pregnant or lactating women were also excluded.

### Procedures

Eligible patients were scheduled for six cycles (every 3 weeks per cycle) of PLD (40 mg/m^2^; CSPC Ouyi Pharmaceutical Co., Ltd., Shijiazhuang, China) plus docetaxel (75 mg/m^2^) and trastuzumab (loading dose 8 mg/kg, maintenance dose 6 mg/kg) intravenously for neoadjuvant treatment. Dose adjustment of PLD from 40 to 35 mg/m^2^ would be performed once grade ≥3 adverse events (AEs) occurred. In addition, discontinuation and suspension of neoadjuvant treatment were allowed when patients experienced disease progression or unacceptable toxicity. Breast-conserving operation or modified radical mastectomy was scheduled within 3–4 weeks after the final dose of chemotherapy based on the condition of patients and their own choice. The decision of postoperative therapy was based on physician preference.

Based on RECIST 1.1, tumor response was assessed by the investigator using magnetic resonance imaging (MRI) every two NAC cycles. Toxicity was assessed by laboratory tests, electrocardiogram (ECG) examination, and intracoronary Doppler ultrasound every one chemotherapy cycle. All eligible patients were followed up until the withdrawal of consent or death.

Before neoadjuvant treatment, ultrasound-guided needle biopsy of the primary tumor in both breast and abnormal lymph nodes was performed for histological diagnosis, including an evaluation of hormone receptors (HRs), HER2, Ki-67, *metadherin (MTDH), glutaminyl-peptide cyclotransferase (QPCT), topoisomerase II alpha (TOP2A)*, and *programmed death ligand 1 (PD-L1). Tumors with estrogen receptor (ER)* or *progesterone receptor (PR)* expression ≥1% were considered as HR-positive. HER2 positivity was defined as immunohistochemistry (IHC) 3+ or 2+ with fluorescence *in situ* hybridization (FISH) positivity. HR, HER2, and Ki-67 status were confirmed at the local pathology department in each research center. The expression of MTDH was assessed based on the staining intensity (0, negative; 1, weak; 2, moderate; 3, strong) and percentage of positively stained tumor cells (0, none; 1, 1%–20%; 2, 21%–50%; 3, 51%–70%; 4, >70%). The immunoreactive score (IRS) was calculated by multiplying the percentage of positively stained tumor cells and staining intensity score. IRS exhibiting ≤4 was regarded as low expression and >4 as high expression. The evaluation criteria for QPCT expression were in accordance with MTDH, except for IRS ≤3 representing low expression. TOP2A expression ≥10% was defined as TOP2A-positive. PD-L1 expression (22C3 antibody) was assessed as the combined positive score (CPS), which was defined as the number of PD-L1-positive cells (tumor cells, lymphocytes, and macrophages) of any type divided by the total number of tumor cells (24). CPS ≥1 was defined as PD-L1-positive. Infiltration status of tumor-infiltrating lymphocytes (TILs) was analyzed by hematoxylin–eosin (HE) staining and was scored as low (0%–10%), moderate (11%–59%), and high (>60%) (25).

### Endpoints

The primary endpoint was total pathological complete response (tpCR), which was defined as the absence of invasive lesions in the breast and axillary lymph nodes (ypT0/is ypN0). Pathological response status was assessed according to Miller–Payne (MP) grading system. The secondary endpoints were breast pathological complete response (bpCR; ypT0/is, defined as no invasive carcinoma in the breast), objective response rate [ORR; calculated as the proportion of patients achieving a complete response (CR) and a partial response (PR) after the last neoadjuvant treatment], operation rate, breast-conserving surgery rate, and safety. Safety was assessed using the Common Terminology Criteria for Adverse Events (CTCAE) version 5.0. Cardiotoxicity was defined as a resting LVEF less than 50%, LVEF that decreased by ≥10% from baseline, or occurrence of congestive heart failure (CHF). Exploratory endpoint was the tpCR according to MTDH, QPCT, TOP2A, and PD-L1 expressions, as well as infiltration status of TILs.

### Statistical Analysis

Sample size of this study was not statistically calculated but was expected to provide sufficient data to support the research purposes. All statistical analyses were performed using IBM SPSS 25.0 (IBM Corp., Armonk, NY, USA). Efficacy analysis was carried out according to full analysis set (FAS), which was defined as all participants who received at least one cycle of neoadjuvant treatment and without serious violation of the eligibility criteria. Safety was evaluated based on safety analysis set (SAS), which was defined as all participants who received at least one cycle of neoadjuvant treatment and at least one assessment of safety data. Categorical variables were presented as percentages and numbers. The proportion of patients with tpCR, bpCR, and ORR was tested and recorded with 95% confidence interval (CI) obtained by the Clopper–Pearson method. Differences between the groups were estimated using chi-square test. A *p* < 0.05 was considered to be statistically significant.

## Results

### Patient Characteristics

**Figure 1:**
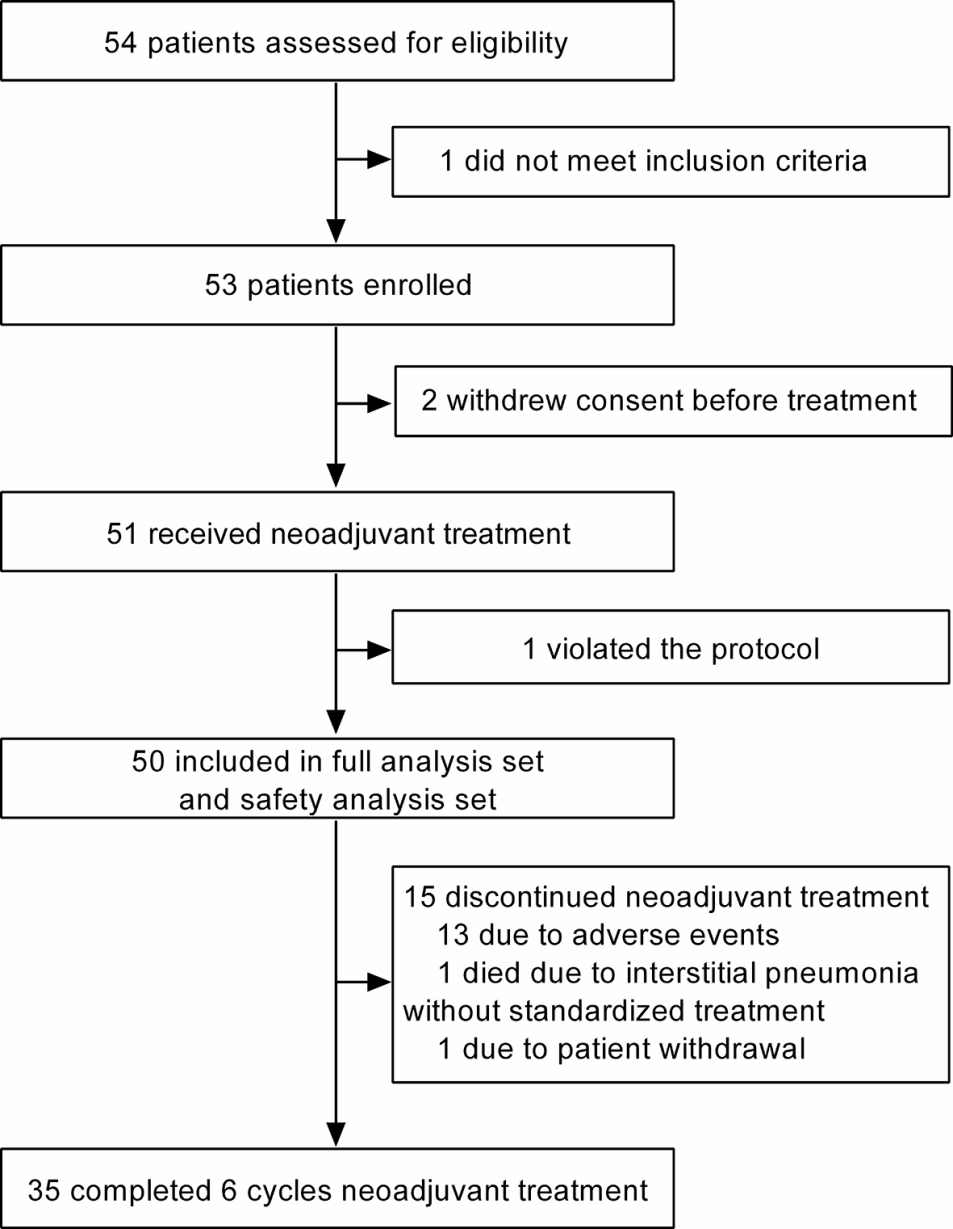
Trial profile.

Between March 2019 and February 2021, 54 patients from 3 centers in China were assessed for eligibility, and 53 were eligible for this trial (one did not meet inclusion criteria). Of the 53 eligible patients, 51 received neoadjuvant treatment (two withdrew consent before treatment), and 50 were finally included in the FAS and SAS **(** **Figure 1****)**. The reason for not being included in the FAS and SAS was protocol violation (n = 1).

Baseline characteristics of the 50 patients are listed in **Table 1**. The median age of the 50 patients was 50.5 years (range, 44.0–56.0 years). The majority of patients were premenopausal (27/50, 54.0%), older than 50 years of age (26/50, 52.0%), had T2 tumors (34/50, 68.0%), and had axillary lymph node involvement (48/50, 96.0%). HR was positive in 58.0% (29/50) of patients, and Ki-67 was >30% in 66.0% (33/50) of patients.

**Table 1:** Baseline characteristics of patients.

	Patients (n = 50)
Age (years), median (range)	50.5 (44.0–56.0)
Age (years; n, %)	
≤50	24 (48.0)
>50	26 (52.0)
Axillary lymph nodes involvement (n, %)	
Positive	48 (96.0)
Negative	2 (4.0)
Menopausal state (n, %)	
Postmenopausal	23 (46.0)
Premenopausal	27 (54.0)
Clinical stage (n, %)	
II	25 (50.0)
III	25 (50.0)
Ki-67 (n, %)	
≤30%	17 (34.0)
>30%	33 (66.0)
Hormone receptor status (n, %)	
Positive	29 (58.0)
Negative	21 (42.0)
Karnofsky performance status (n, %)	
100	36 (72.0)
90	12 (24.0)
80	2 (4.0)
Tumor size stage (n, %)	
T1	5 (10.0)
T2	34 (68.0)
T3	5 (10.0)
T4	6 (12.0)
Surgery (n, %)	
Modified radical mastectomy	27 (54.0)
Breast-conserving surgery	22 (44.0)
Surgery not performed	1 (2.0)

### Clinical Activity

In total, 35 patients (70.0%) completed 6 cycles of neoadjuvant treatment, and 41 patients (82.0%) completed 4–6 cycles of neoadjuvant treatment. Reasons for 15 cases that did not complete 6 cycles of neoadjuvant treatment included intolerable AEs (n = 13), death due to interstitial pneumonia without standardized treatment for the AE (n = 1), and patient withdrawal because of allergy to granulocyte colony-stimulating factor (n = 1). Of these, the AEs that led to incompletion of the full course regimen were grade 2–4 hand–foot syndrome (HFS) (6 cases, 12%), grade 3 interstitial pneumonia (4 cases, 8%), grade 2 stomatitis (2 cases, 4%), and intolerable grade 2 malaise (2 cases, 4%).

Forty-nine (98.0%) of 50 patients received surgery after neoadjuvant treatment finally, of which 22 (44.0%) underwent breast-conserving surgery and 27 (54.0%) underwent modified radical mastectomy. The reason for one surgery cancelation was death due to interstitial pneumonia without standardized treatment. The median interval from the completion of neoadjuvant therapy to surgery was 3.1 weeks (interquartile range, 2.9–4.9 weeks).

Twenty-four (48.0%; 95% CI, 33.7%–62.6%) of 50 patients achieved a tpCR, and 30 (60.0%; 95% CI, 45.2%–73.6%) had a bpCR. The univariate analyses revealed that age, menopausal state, tumor size stage, axillary lymph node involvement, clinical stage, or Ki-67 level was not associated with tpCR (all *p* > 0.05; **Table 2**) or bpCR (all *p* > 0.05). HR status was associated with bpCR (HR-negative vs. HR-positive: 76.2% vs. 48.3%, *p* = 0.047) but not tpCR (*p* > 0.05).

**Table 2:** The effect of clinical characteristic variables on the tpCR.

	tpCR (n = 50)		*p* value
	Yes (n = 24)	No (n = 26)	
Age			0.402
≤50	13 (54.2)	11 (45.8)	
>50	11 (42.3)	15 (57.7)	
Menopausal state			0.982
Postmenopausal	11 (47.8)	12 (52.2)	
Premenopausal	13 (48.1)	14 (51.9)	
Axillary lymph nodes involvement			0.506
Positive	24 (50.0)	24 (50.0)	
Negative	0 (0.0)	2 (100.0)	
Clinical stage			0.571
II	11 (44.0)	14 (56.0)	
III	13 (52.0)	12 (48.0)	
Ki-67			0.402
≤30%	7 (41.2)	10 (58.8)	
>30%	17 (51.5)	16 (48.5)	
Hormone receptor status			0.271
Positive	12 (41.4)	17 (58.6)	
Negative	12 (57.1)	9 (42.9)	
Tumor size stage			0.459
T1	2 (40.0)	3 (60.0)	
T2	17 (50.0)	17 (50.0)	
T3	1 (20.0)	4 (80.0)	
T4	4 (66.7)	2 (33.3)	

tpCR, total pathological complete response.

Of the 50 patients, 19 (38.0%) achieved a CR, 23 (46.0%) achieved a PR, and 6 (12.0%) had stable disease (SD) as their best response, for an ORR of 84.0% (95% CI, 70.9%–92.8%). No patient experienced disease progression.

### Exploratory Endpoint

**Figure 2:**
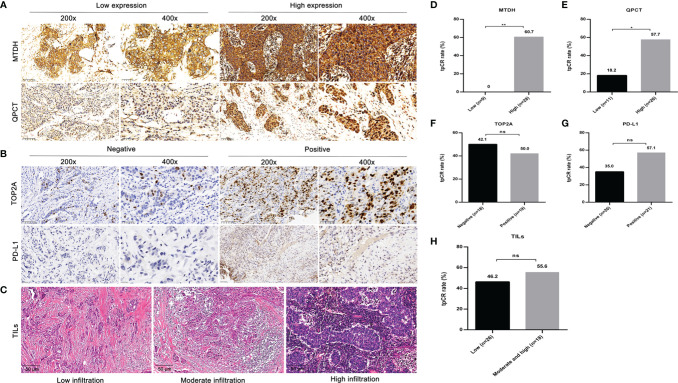
The representative images and effect of MTDH, QPCT, TOP2A, and PD-L1 expressions, as well as infiltration status of TILs on tpCR. **(A)** Representative IHC images of MTDH and QPCT (low and high expressions). **(B)** Representative IHC images of TOP2A and PD-L1 (positive and negative). **(C)** Representative HE staining images of TILs. **(D)** The tpCR according to MTDH expressions. **(E)** The tpCR according to QPCT expressions. **(F)** The tpCR according to TOP2A expressions. **(G)** The tpCR according to PD-L1 expressions. **(H)** The tpCR according to infiltration status of TILs. tpCR, total pathological complete response; MTDH, metadherin; QPCT, glutaminyl-peptide cyclotransferase; TOP2A, topoisomerase II alpha; PD-L1, programmed death ligand 1; TILs, tumor-infiltrating lymphocytes; IHC, immunohistochemistry; HE, hematoxylin–eosin. ^*^ *p* < 0.05; ^**^ *p* < 0.01.; ns, no significance.

The IHC results of MTDH, QPCT, and TOP2A were available in 37 cases. High expression of MTDH and QPCT was detected in 28 (75.7%) and 26 (70.3%) patients, respectively. The incidence of negative and positive expression for TOP2A was exhibited similarly (48.6% vs. 51.4%). Both MTDH and QPCT were highly expressed in 59.5% (22/37) of 37 cases. Patients who carried a high level of MTDH (60.7%, *p* = 0.002; **Figures 2A, D****)** or QPCT (57.7%, *p* = 0.036; **Figures 2A, E****)** or both (MTDH_high_QPCT_high_, 68.2%; *p* = 0.002) were inclined to achieve a tpCR. There was no significant difference in tpCR between patients with TOP2A negativity and TOP2A positivity (*p* = 0.75; **Figures 2B, F****)**.

The PD-L1 expression was assessed in 41 cases. Although patients with PD-L1 positivity (12/21, 57.1%) tended to show a higher tpCR proportion compared to those with PD-L1 negativity (7/20, 35.0%), there was no significant difference (*p* = 0.155; **Figures 2B, G****)**.

Due to the limited amount of puncture tissue, 44 cases had available TIL results. Among them, 26 (59.1%) exhibited low infiltration, 17 (38.6%) displayed moderate infiltration, and 1 (2.3%) showed high infiltration. The tpCR of patients with low infiltration status of TILs (46.2%) was lower than those with moderate and high infiltration status (55.6%), although without statistical significance (*p* = 0.76; **Figures 2C, H****)**.

### Safety

Treatment-related AEs **(** **Table 3****)** of any grade occurred in 88.0% (44/50) of all patients, and most AEs were grades 1–2. In this study, 38.0% (19/50) of cases experienced grade ≥3 AEs. The most common AEs were oral mucositis (68.0%), followed by HFS (56.0%) and watery eyes (40.0%). Nineteen patients (38.0%) experienced dose reduction of PLD due to grade 3 AEs including oral mucositis and HFS. One patient died of neoadjuvant treatment-related interstitial pneumonia without standardized treatment for the AE.

**Table 3:** Treatment-related adverse events in all patients (n = 50).

Adverse events (n, %)	Grade 1–2	Grade 3–4	Any grade
Hematological toxicity			
Leukopenia	7 (14.0)	6 (12.0)	13 (26.0)
Neutrocytopenia	1 (2.0)	3 (6.0)	4 (8.0)
Non-hematological toxicity			
Oral mucositis	30 (60.0)	4 (8.0)	34 (68.0)
Hand–foot syndrome	22 (44.0)	6 (12.0)	28 (56.0)
Watery eyes	20 (40.0)	0 (0.0)	20 (40.0)
Diarrhea	16 (32.0)	3 (6.0)	19 (38.0)
Alopecia	18 (36.0)	0 (0)	18 (36.0)
Nausea	17 (34.0)	0 (0)	17 (34.0)
Vomiting	13 (26.0)	0 (0)	13 (26.0)
Anorexia	12 (24.0)	0 (0)	12 (24.0)
Fever	11(22.0)	0 (0)	11 (22.0)
Malaise	11 (22.0)	0 (0)	11 (22.0)
LVEF reduction	9 (18.0)	0 (0.0)	9 (18.0)
Bloating	9 (18.0)	0 (0)	9 (18.0)
Insomnia	6 (12.0)	2 (4.0)	8 (16.0)
Headache	8 (16.0)	0 (0)	8 (16.0)
Cough	6 (12.0)	1 (2.0)	7 (14.0)
Dysphagia	5 (10.0)	1 (2.0)	6 (12.0)
Alanine aminotransferase elevation	6 (12.0)	0 (0)	6 (12.0)
Interstitial pneumonia	0 (0)	5 (10.0)	5 (10.0)
Constipation	5 (10.0)	0 (0)	5 (10.0)
Palpitations	5 (10.0)	0 (0)	5 (10.0)
Oropharyngeal pain	3 (6.0)	0 (0)	3 (6.0)
Aspartate aminotransferase elevation	4 (8.0)	0 (0)	4 (8.0)
Cutaneous pigmentation	3 (6.0)	0 (0)	3 (6.0)
Sore throat	3 (6.0)	0 (0)	3 (6.0)
Ventricular premature contraction	1 (2.0)	0 (0)	1 (2.0)

LVEF, left ventricular ejection fraction.

**Figure 3:**
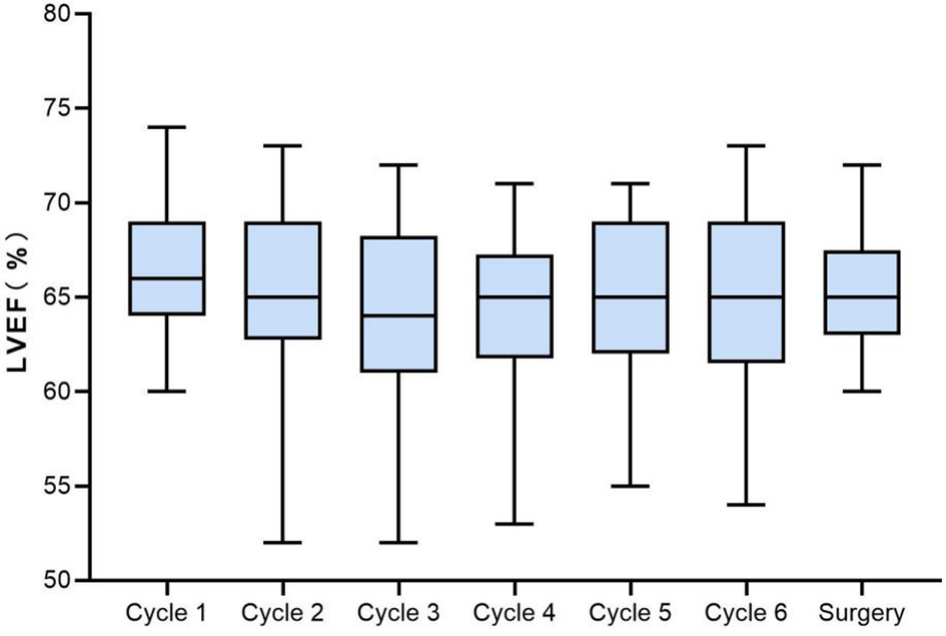
Boxplots of LVEF from baseline to surgery in the assessable population. LVEF, left ventricular ejection fraction.

From baseline to surgery, LVEF decline by ≥10% was noted in 9 patients (18%), but none of them was <50%. The changes of LVEF in all assessable patients are displayed in **Figure 3**. LVEF decline was observed initially after 1 cycle of NAC in one case, while most of the LVEF reduction events occurred after 3 cycles of chemotherapy. Six of the 9 decreased LVEF were recoverable, and 2 of them ultimately recovered to baseline level after a full course of neoadjuvant therapy. CHF was not noted in this study. Apart from LVEF reduction, palpitation (grade 1 or 2) was observed in 10.0% (5/50) of patients. Grade 1 ventricular premature contraction was reported in only 1 case before the second cycle of neoadjuvant treatment.

## Discussion

To the best of our knowledge, this multicenter, open-label, single-arm, phase II study provided the first analysis of the combination of PLD, trastuzumab, and docetaxel for the neoadjuvant treatment of patients with HER2-positive BC. In general, most of the patients (70.0%) completed the full course (6 cycles) of NAC, and relatively high tpCR (48%) and bpCR (60%) were attained in the present study, which demonstrated that PLD plus docetaxel and trastuzumab might be a potential neoadjuvant regimen for HER2-positive BC.

The achievement of pCR after neoadjuvant treatment has been confirmed to be associated with better long-term outcomes in terms of progression-free survival and overall survival (26–28). Accordingly, tpCR and bpCR were defined as efficacy endpoints in our study. PLD plus docetaxel and trastuzumab in the neoadjuvant setting for stage II or III HER2-positive BC showed good antitumor activity, with a tpCR rate of 48.0% and a bpCR rate of 60.0%. The tpCR obtained in our study was higher than that observed in other studies (6, 19, 20). Uriarte-Pinto et al. (6) demonstrated that the tpCR rate of patients with HER2-positive BC managed with LD plus trastuzumab and paclitaxel was 40%. Besides, two phase II clinical trials evaluated the efficacy of LD in combination with trastuzumab and docetaxel as neoadjuvant treatment for patients with HER2-positive BC and reported tpCR rates of 27% (19) and 38.3% (20). The patient characteristics including clinical stage, Ki-67 level, and HR status, as well as different chemotherapeutic regimens, might contribute to this discrepancy in pCR. It was worth noting that the pCR observed in our study was even comparable to double anti-HER2 therapy (29). The Opti-HER HEART trial documented a tpCR rate of 56.6% following neoadjuvant LD, paclitaxel, trastuzumab, and pertuzumab in HER2-positive BC (29). In addition, patients with early-stage HER2-positive BC in a real-world study presented a tpCR rate of 54% after neoadjuvant pertuzumab and trastuzumab with chemotherapy (30). However, pertuzumab is not available in some regions for a variety of reasons; thus, single trastuzumab plus chemotherapy remains to be the preferred regimen. In addition, the ORR and breast-conserving surgery rates observed in our study were 84.0% and 44.0%, respectively. Taken together, the neoadjuvant regimen containing PLD plus docetaxel and trastuzumab was active for patients with HER2-positive BC. However, further follow-up should be conducted to verify whether the high pCR rate could contribute to the survival benefit.

Accumulating evidence had illustrated that HR-negative cases had the advantage of achieving pCR (31, 32). Our data did not observe the variance between HR-positive and HR-negative subsets as for tpCR. However, patients who had HR-negative status were more likely to achieve bpCR compared with those who had HR-positive status (76.2% vs. 48.3%, *p* = 0.047). Prior findings discovered that patients with high Ki-67 level, small tumor size, or low clinical stage have better access to get pCR (33–35). Due to the limited sample size, this study failed to confirm the correlation between tpCR and other clinicopathological indicators. Thus, studies with large sample sizes are necessary to further investigate predictions of pCR and prognosis.

MTDH acting as an oncogene can promote tumor cell proliferation and inhibit apoptosis (36). Overexpression of MTDH is noted in aggressive BC subsets, such as triple-negative BC and HER2-overexpressed tumors (37). QPCT, a secreted protein implicated in the biosynthesis of pyroglutamyl peptides, is found to contribute to angiogenesis (38). Our preceding experiment suggested that MTDH and QPCT were intensively expressed in local advanced breast tumors and positively correlated with poor disease-free survival. The current study showed that patients who carried a high level of MTDH, QPCT, or both were more liable to attain tpCR. Interestingly, published studies demonstrated that a high expression of MTDH was associated with drug resistance (39), but our cases with a high expression of MTDH showed a sensitive response to neoadjuvant treatment. Of note, we only examined the MTDH level at baseline, not after neoadjuvant therapy, which may be not enough to explain the relationship between MTDH and drug resistance. The changes of MTDH after neoadjuvant treatment should be probably taken into consideration to evaluate its correlation with chemoresistance. Although pCR after preoperative therapy is considered to be a powerful surrogate of survival, in view of the influence of MTDH on chemoresistance and trastuzumab resistance, follow-up should be continued to further assess the influence of MTDH on long-term efficiency.

TOP2A is a proliferation marker associated with Ki-67 index and tumor grade (40). Considering that tumors with TOP2A positivity were more sensitive to anthracyclines (41), the expression TOP2A was tested in our study. However, there was no trend to obtain pCR in cases with TOP2A positivity, which might be attributed to the limited sample size and different interpretation criteria for TOP2A. Evidence has shown that PD-L1 positivity is correlated with a high proportion of pCR rate in patients with HER2-positive BC (42). In our study, patients with PD-L1 positivity tended to show a higher tpCR proportion compared to those with PD-L1 negativity, but statistical significance was not reached in tpCR due to the small sample size. It is documented that the high infiltration status of TILs before neoadjuvant treatment can significantly predict a high pCR rate of HER2-positive BC (25). Nevertheless, only 1 (2.3%) case exhibited high infiltration of TILs in our study due to the small sample size. Thus, we failed to observe a significant trend to achieve pCR in patients with a high infiltration status of TILs.

Although our studied treatment attained excellent efficiency, the safety issues related to treatment deserve attention. First of all, some patients did not complete the full course of chemotherapy due to poor tolerance. The completion rate of NAC in terms of concomitant use of anthracyclines, taxanes, and trastuzumab in published clinical trials differs. In the GEICAM 2003-03 study (43), only 4 patients (6%) did not finish the full course of NAC schedule containing liposome-encapsulated doxorubicin (50 mg/m^2^) and docetaxel (60 mg/m^2^) on day 1 every 3 weeks combined with trastuzumab (4 mg/kg loading dose on day 1, followed by 2 mg/kg weekly). Gavilá et al. (44) reported that 72.6% of the NAC settings completed a full course of study treatment with six cycles of non-pegylated liposome-encapsulated doxorubicin (50 mg/m^2^ every 3 weeks), paclitaxel (80 mg/m^2^ weekly), and trastuzumab (loading dose 4 mg/kg, maintenance dose 2 mg/kg weekly). In another observational study (6), 66.7% of the studied patients finished six cycles of the combination of non-pegylated liposome-encapsulated doxorubicin (50 mg/m^2^ on day 1 every 3 weeks), paclitaxel (80 mg/m^2^ on days 1, 7, and 14), and trastuzumab (loading dose 8 mg/kg, maintenance dose 6 mg/kg every 3 weeks). Accordingly, considering the variation in drug type and initial drug dosage, our therapeutic regimen has been relatively well tolerated. Secondly, consistent with previous reports (45, 46), the most frequent PLD-related AEs of any grade in our study were oral mucositis (68.0%) and HFS (56.0%), and most AEs were grades 1–2. These AEs were the main cause for the incompleted full course of chemotherapy, which may result from the high dosage of PLD (40 mg/m^2^–35 mg/m^2^) relative to that of other studies (35 mg/m^2^–30 mg/m^2^). In one of our published studies (47), four cycles of PLD (40 mg/m^2^) plus cyclophosphamide (600 mg/m^2^) on day 1 of a 21-day schedule, followed by four cycles of docetaxel (85 mg/m^2^) on day 1 of a 21-day schedule, were tested in NAC populations whatever the molecular typing. Although 86.6% of all cohorts completed the full course of chemo, the pCR was lower than that of the current data. Even if the incidence of HFS (45.53%) and oral mucositis (39.28%) was relatively low, nausea/vomiting (81.25%) and fatigue (74.11%) were the most common events. The mechanism underlying skin and mucous injury induced by PLD was not fully elucidated, but it might be related to increased vascular permeability caused by PLD (48). Our team is dedicating to explore potential approaches to cope with HFS caused by PLD, and we have reported that calcium dobesilate (CaD) could alleviate HFS in the Sprague–Dawley rat model treated by PLD (49). The clinical trial on the prevention of HFS by CaD conducted in PLD-treated BC settings is undergoing, and the result will be published in the future. In the present study, only one case died of interstitial pneumonia after 3 cycles of neoadjuvant treatment, which mainly resulted from the lack of standardized treatment for the AE.

It has been reported that traditional anthracyclines (doxorubicin and epirubicin) combined with trastuzumab might cause cardiotoxicity (11). To avoid potential heart problems, PLD was used as a substitute for traditional anthracycline. In a series of studies assessing concurrent use of trastuzumab and PLD for metastatic HER2-positive BC, around 4.5%–23% of LVEF decline was noted (50–52). The diversity may be on account of different therapeutic regimens and characteristics of patients. In the present study, PLD in combination with trastuzumab showed good cardiac safety, with 9 (18.0%) cases that experienced LVEF decline by ≥10%, 2 of which recovered to baseline before surgery. No case of CHF was observed, and no death from cardiotoxicity occurred in our study. Follow-up will be carried out to monitor long-term effects of PLD plus trastuzumab on cardiac function. To sum up, PLD in combination with docetaxel and trastuzumab was generally well tolerated for HER2-positive BC in the neoadjuvant setting.

In consideration of the overlap in cardiotoxicity of anthracyclines and trastuzumab, clinical trials have attempted therapeutic protocols in the absence of anthracyclines. However, there is no unified conclusion until now. In terms of efficacy, the PH-FECH (paclitaxel 80 mg/m2 weekly for 12 weeks or paclitaxel 225 mg/m2 every 3 weeks, followed by 4 cycles of FEC (fluorouracil 500 mg/m2, epirubicin 75 mg/m2, and cyclophosphamide 500 mg/m2 ) on day 1, and trastuzumab loading dose 8 mg/kg, maintenance dose 6 mg/ kg, every 3 weeks regimen showed a higher pCR rate (60.6% vs. 43.3%) and relapse-free survival (RFS) advantage (93% vs. 71%) compared with TCH (docetaxel 75 mg/m2 IV on day 1, carboplatin at an area under the concentration curve (AUC) of 6 IV on day1, and trastuzumab loading dose 8 mg/kg, maintenance dose 6 mg/ kg, administered at 3-week intervals for 6 cycles in a retrospective study (53). Even when the dual HER2 blockade was applied, the pCR did not differ significantly [TRYPHAENA (54): anthracycline group vs. non-anthracycline: 61.6% vs. 66.2%, TRAIN-2 (55): anthracycline group vs. non-anthracycline: 67% vs. 68%]. As for the safety, the combination of trastuzumab and anthracycline did not result in significant difference in cardiotoxicity (53–55). However, grade 3 or worse febrile neutropenia was the most common AE in anthracycline-included regimens (53–55). Consequently, we selected PLD in our study to alleviate the cardiotoxicity and hematologic toxicity. Although in the era of optimized HER2-directed therapies trastuzumab concurrent with anthracycline and taxanes (ATH) is not the preferred strategy in the guidelines, ATH might be an alternative option in HER2-positive NAC setting if dual blockade with pertuzumab and trastuzumab is not available.

There were some limitations in the study. Firstly, this trial was limited by the small sample size of patients who were only recruited from 3 hospitals in China. The results might not be generalized to BC cases from other geographic regions or other racial or ethnic backgrounds. Secondly, due to the combination of PLD plus trastuzumab and docetaxel, it was impossible to ascertain the respective contribution of each drug to the overall therapeutic effect. Nonetheless, our results showed that this drug combination appeared to be active for patients with HER2-positive BC with acceptable safety. Thirdly, the open-label, single-arm study design precluded the comparison of clinical benefits with traditional anthracyclines. Finally, due to the relatively short follow-up period, the long-term efficacy as progression-free survival and overall survival was not mature. Thus, randomized controlled trials with larger sample sizes and longer follow-up time are necessary in the future to continue to validate the efficacy and safety of PLD plus docetaxel and trastuzumab for the neoadjuvant treatment of patients with HER2-positive BC.

## Conclusions

In conclusion, the first published phase II study demonstrated that the neoadjuvant regimen containing PLD plus docetaxel and trastuzumab showed good antitumor activity for patients with stage II or III HER2-positive BC, with a relatively high tpCR and bpCR rate. The majority of AEs related to this regimen were mild and controllable, with an acceptable cardiotoxicity profile. These findings suggest that this neoadjuvant regimen might offer a potential therapeutic option for HER2-positive BC. Further follow-up is still needed to confirm the long-term benefit of the neoadjuvant regimen for this population. MTDH and QPCT in BC tissues pre-neoadjuvant are potential predictive markers for tpCR.

## Data Availability Statement

The original contributions presented in the study are included in the article/**Supplementary Material**. Further inquiries can be directed to the corresponding authors.

## Ethics Statement

The studies involving human participants were reviewed and approved by the ethics committee of the Fourth Hospital of Hebei Medical University. The patients/participants provided their written informed consent to participate in this study.

## Author Contributions

ZS conceived and designed the study. YTL, YQ, EZ, XK, and CY provided study materials and performed data collection. HW, QY, and CZ were involved in data analysis and interpretation and article writing. YPL examined the pathology and immunohistochemistry. All authors were involved in final approval of the article.

## Funding

This study was supported by the Natural Science Foundation of Hebei Province (H2020206365, H2021206071), Special Fund for Clinical Research of Wu Jieping Medical Foundation (320.6750.2020-07-17), and Beijing Xisike Clinical Oncology Research Foundation (Y-SY201901-0021).

## Conflict of Interest

The authors declare that the research was conducted in the absence of any commercial or financial relationships that could be construed as a potential conflict of interest.

## Publisher’s Note

All claims expressed in this article are solely those of the authors and do not necessarily represent those of their affiliated organizations, or those of the publisher, the editors and the reviewers. Any product that may be evaluated in this article, or claim that may be made by its manufacturer, is not guaranteed or endorsed by the publisher.
